# Curative use of forequarter amputation for recurrent breast cancer over an axillary area: a case report and literature review

**DOI:** 10.1186/1477-7819-12-346

**Published:** 2014-11-18

**Authors:** Chun-Hao Tsai, Huey-En Tzeng, Wei-Kae Juang, Pei-Guo Chu, Patricia Fann, Yi-Chin Fong, Horng-Chaung Hsu, Yun Yen

**Affiliations:** Department of Orthopedics, China Medical University Hospital, No. 91 Hsueh-Shih Road, Taichung, 404 Taiwan; Graduate Institute of Clinical Medical Science, China Medical University, No. 91 Hsueh-Shih Road, Taichung, 404 Taiwan; Division of Hematology/Oncology, Taichung Veterans General Hospital, 1650 Taiwan Boulevard Sect. 4, Taichung, 40705 Taiwan; Taipei Medical University, 250 Wuxing Street, Taipei City, 110 Taiwan; Department of Pathology, City of Hope National Medical Center, 1500 East Duarte Road, Duarte, CA 91010 USA; Department of Medical Oncology, City of Hope National Medical Center, 1500 East Duarte Road, Duarte, CA 91010 USA; School of Chinese Medicine, China Medical University, No. 91 Hsueh-Shih Road, Taichung, 404 Taiwan; Department of Molecular Pharmacology, City of Hope National Medical Center and Beckman Research Center, 1500 East Duarte Road, Duarte, CA 91010 USA; The PhD Program for Cancer Biology and Drug Discovery, Taipei Medical University, 250 Wuxing Street, Taipei City, 110 Taiwan

**Keywords:** Breast cancer, Forequarter amputation, Recurrence

## Abstract

Axillary recurrence of breast cancer that involves the brachial neurovascular bundle is uncommon. However, for many patients with such recurrence, forequarter amputation can play a palliative role in relieving excruciating pain and paralysis of the upper limb. Further, for those patients who do not have distant metastasis or other local-regional recurrence, forequarter amputation provides a chance for a cure. Only a few case reports of curative amputations for recurrent breast cancer are present in the literature. Here, we report a case of forequarter amputation for curative treatment of axillary recurrent breast cancer, together with a literature review. To date, we have followed the patient for three years after amputation, during which there has been no evidence of recurrence or metastasis. Although radical resection is feasible, it can be accompanied by surgical wound complications and psychosocial stress. Therefore, an organized multidisciplinary approach is needed to ensure the success of radical resection.

## Background

As medical treatment of breast cancer has improved, radical amputation as a means of treatment is less often performed. Forequarter or interscapulothoracic amputation involves removing the entire upper extremity, including the ipsilateral scapula and clavicle. Use of forequarter amputation to treat recurrent breast cancer was first reported in 1900 by Buchanan [1], and four additional cases were reported by Pack in 1942 [2]. Since then, few instances of forequarter amputation for the surgical management of recurrent breast cancer have been reported. Indeed, to date, including the cases mentioned above, only 23 cases of forequarter amputation to treat recurrent breast cancer have been described in the literature, and only five of these amputations were performed primarily for curative purposes [3–6]. Here, we report the case of a patient with metastatic breast adenocarcinoma who underwent forequarter amputation.

## Case presentation

**Figure 1:**
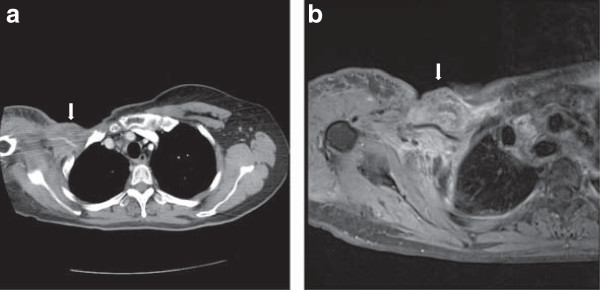
**Preoperative coronal CT (a) and T1-weighted MRI (b) images of the tumor lesion surrounding the brachial plexus over the right axillary area with chest wall invasion (arrow).**

In 2002, a 52-year-old asian woman underwent a lumpectomy for a stage II infiltrating duct carcinoma over her right breast, followed by four cycles of adjuvant chemotherapy combining doxorubicin, cyclophosphamide and paclitaxel. Chemotherapy was followed by locoregional radiation. Eight years after the completion of chemotherapy, she found a slowly growing mass on her right neck. Positron emission tomography (PET) scan revealed large infraclavicular mass and a supraclavicular adenopathy of the right neck and right nasal cavity. Analysis of biopsy samples from both masses was consistent with metastatic carcinoma and suggestive of the metastasis having arisen from primary breast cancer. She underwent removal of the right supraclavicular and infraclavicular masses at that time, followed by adjuvant chemotherapy. However, one year after the surgery, she found a mass in the upper outer quadrant of her right anterior chest wall. Ulceration and bleeding of the mass caused her to have a painful disability of her right upper limb. A computed tomography (CT) scan (Figure 1a) revealed a mass in the upper outer quadrant of her right anterior chest wall. The mass had invaded the ribs and pleura. Magnetic resonance imaging (MRI, Figure 1b) revealed that the mass involved the axillary artery and occluded the right lateral subclavian vein and axillary vein.

After a multimodality assessment followed by discussion of the available options and risks, benefits and subsequent rehabilitation associated with forequarter amputation with the patient and her family, she consented to the amputation. Surgery revealed a fungating tumor in the anterior superior chest wall that involved the right first, second and third ribs. This tumor had adhered to the right lung as well as the brachiocephalic-subclavian vein and artery.

**Figure 2:**
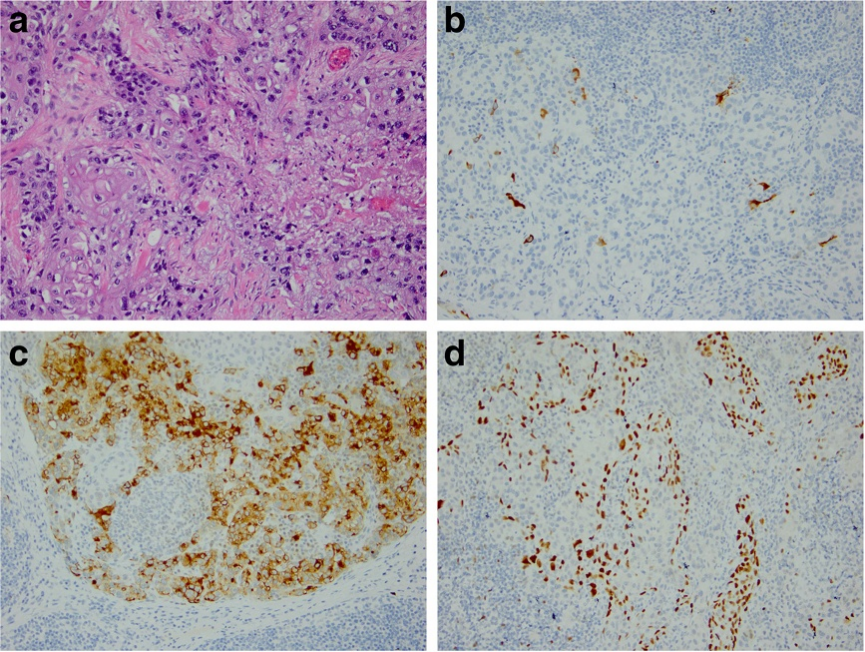
**Micrographs of the samples came from the same block proved metastatic breast carcinoma. (a)** Hematoxylin and eosin staining shows intracytoplasmic mucin and glandular formation. Immunohistochemical staining for **(b)** mammaglobin, **(c)** gross cystic disease fluid protein 15 and **(d)** P63. All images 200x magnification.

During surgery, the patient was placed in a semi-lateral position on her left side with her right side elevated at a 45° angle. An incision was made over her sternocleidomastoid muscle, about 3 cm above where the muscle attaches to the clavicle. The incision was directed posteriorly around the posterior triangle of the neck, around the scapula, under the axilla(including an extension along the latissimus dorsi muscle to allow for proper flaps) and then anteriorly around the mass and across the chest wall. Subcutaneous dissection was done along the anterior border of the manubrium and the right first, second and third ribs. For the scapulectomy, an incision was made in the patient’s back, and the teres major and latissimus dorsi were dissected to obtain negative margins. The latissimus dorsi muscle was completely transected from the chest wall and scapula and a flap was created under the teres major. The intercostal continuous dissection was carried up to the neck. The platysma muscle was divided, and the sternocleidomastoid muscle identified and divided to identify the jugular vein, carotid artery and vagus nerve. Once the neck musculature was divided and the carotid sheath contents isolated, the posterior scalene muscle was clearly identified. Then the posterior scalene muscles were divided sequentially while the brachial plexus branches were identified. At that time, the phrenic nerve was traced and dissected from the tumor, and the brachiocephalic vein and internal jugular vein that had adhered to the tumor were ligated. The remaining attachments of the structures to the tumor and arm were dissected, including the subclavian artery and first ribs. Then the chest wall was covered and reconstructed with the free forearm flap. Histological analysis of the metastatic mass showed features of primary breast cancer of intracytoplasmic mucin and glandular formation in hematoxylin and eosin staining (Figure 2a). Immunohistochemical staining revealed apositive finding of mammaglobin (Figure 2b), gross cystic disease fluid protein 15 (Figure 2c) and P63 (Figure 2d).

**Figure 3:**
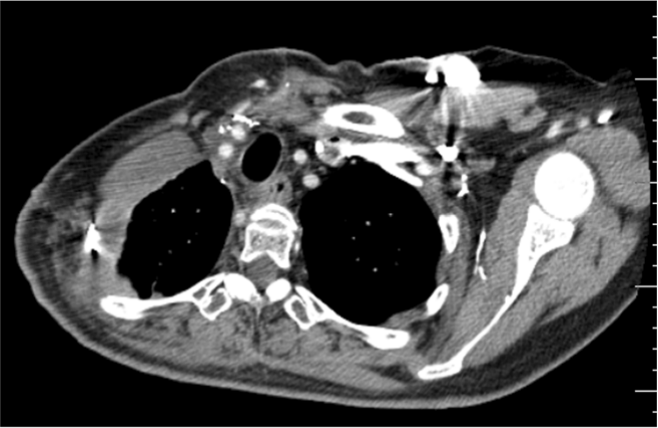
**Coronal CT taken three years after surgery.** There is no evidence of recurrence or invasion of malignancy.

The patient experienced no wound complications during follow-up after surgery. To date, she has been followed up on for three years after surgery and has shown no signs of malignancy (Figure 3).

## Discussion

Breast cancer is one of the leading causes of death in women [7]. Despite advances in systemic adjuvant therapy that have led to increased survival rates of women with breast cancer [8], recurrent breast cancer is still frequently lethal [9]. Approximately 5 to10% of patients who undergo mastectomy for operable breast cancer will develop a chest wall or regional nodal recurrence within 10 years after surgery [10]. There is wide variability in the reported incidence of simultaneous locoregional and distant metastases (10 to 60%) [10–16]. However, approximately one-third of patients who present with a post-mastectomy locoregional recurrence have synchronous distant disease [10].

Axillary recurrence is uncommon [17, 18], and its symptoms are often initially vague. The initial involvement of the brachial plexus may be misinterpreted as a radiation- or chemotherapy-induced brachial plexopathy [19]. Delayed diagnosis and treatment of axillary recurrence may lead to tumor progression, which can include extensive involvement of the brachial neurovascular bundle or underlying soft tissues [6, 20]. Invasion of the brachial plexus and axillary vessels causes pain, limb dysfunction, lymphedema, paralysis and sensory impairment. Further, destruction of the overlying soft tissue leads to invasion of the chest wall, skin ulceration, hemorrhage and even bacterial or fungal infections and sepsis.

Decisions regarding therapy for local disease are often affected by whether the patient receives systemic treatment and the response to such therapy. For patients who may need a large resection, systemic therapy is preferable before resection not only to facilitate successful locoregional treatment, but also to eliminate any early metastases. Surgical treatment is useful for controlling pain and ulcers and maintaining the patient’s quality of life [21]. However, sometimes a more radical surgical intervention, such as amputation, is recommended in order to improve the patient’s quality of life and ability to perform daily functions [20, 22–24].

Although the majority of forequarter amputations are performed for high-grade bone and soft tissue sarcomas or extensive osteomyelitis of the upper extremities, this radical operation may also be recommendedfor the curative treatment of recurrent breast cancer or the palliation of locally advanced breast cancer [3–6, 20, 24–27]. The feasibility of completely reducing tumor burden through the use of wide excision depends on the degree of tumor invasion to neighboring vital organs. If the tumor involves the chest wall, an extended forequarter amputation could be performed, including resection of part of the chest wall [6, 28, 29], sometimes in combination with a pneumonectomy [30–32]. In most patients, primary wound closure is achieved [33]. However, soft tissue reconstruction is requiredfor large defects that cannot achieve primary closure. For softtissue reconstruction various options have been used, such as split skin graft, pedicled omentoplasty [34], fasciocutaneous deltoid flap [33, 35], free filet extremity flap [28] or in one case an osteomyocutaneous free flap that incorporated the elbow joint from the amputated extremity to reconstruct the shoulder contour [36].

Most patients who have needed forequarter amputation have had distant metastasis or other local-regional recurrence, and therefore the amputations were for palliative purposes. Thus, most patients survived less than two years after forequarter amputation [19, 20, 24–27]. However, in cases of solitary lesions that do not have associated comorbidities, forequarter amputation is recommended not only for relief of symptoms, but also for a chance of curative treatment. The literature (Table 1) documents five cases in which forequarter amputation was performed with the goal of a cure; three of these patients lived for at least three years after amputation. However, there was also a higher rate of wound complications (60%, three of five reported cases) when forequarter amputation was used with the goal of a cure because of the wider dissection margin and larger soft-tissue defect. The benefit of postoperative radiation is unclear, however, we do not recommend postoperative radiation in those who have been previously irradiated because of previous poor response and increased potential for wound and/or flap complications [37].

**Table 1:** **Reports of forequarter amputation for curative treatment of axillary recurrence breast cancer**

Reference	Number of patients	Age/Gender	Diagnosis	Indication	Wound complication	Local recurrence	Survival
Pressman [2]	2	55/F	Recurrent	Curative	None delayed healing	None	A (48 months)
		67/F					
							A (36 months)
Sakamura *et al*. [3]	1	57/F	Recurrent	Curative	Flap fringe necrosis	None	D (22 Months)
Goodman *et al*. [5]	1	56/F	Recurrent	Curative	wound care flap necrosis	None	A (35 months)
Ayvaz *et al*. [4]	1	54/M	Recurrent	Curative	Not mentioned	Lung metastasis at 6 months	D (11 months)
Tsai *et al*. [current study]	1	52/F	Recurrent	Curative	None	None	A (36 months)

A: alive; D: dead.

A multidisciplinary approach to breast cancer care is essential to the successful integration of available therapies [38–40]. When forequarter amputation is used for curative treatment, comprehensive treatment is needed throughout preoperative assessment, resection and the postoperative period. The forequarter amputation itself and skin graft, if needed, require a surgical team of orthopedic, general and plastic-reconstructive surgeons, while a team of radiologists, medical oncologists and radiation oncologists are needed for pre and postoperative evaluation and management. A comprehensive pain management team is also critical, not only to manage the patient’s limb pain before surgery, as most patients who require forequarter amputation experience intractable and prolonged neurogenic pain prior to amputation [41, 42], but also the phantom pain after radical amputations. Although significant pain relief is expected after a radial amputation, the literature indicates a 77% incidence of phantom limb pain after major limb amputation [41].

A rehabilitation team, including psychological support, is also essential. Psychological concerns for amputees include fear of the unknown, loss of self-esteem, loss of self-confidence, fear of rejection and distress arising from having a malignant disease with a poor prognosis. This emotional distress can contribute to suicide [43], therefore, counseling is necessary before and after the surgery and psychological support is imperative for successful rehabilitation [44].

## Conclusions

We have reported the rare clinical occurrence of local-regional recurrence of breast cancer over an axillary area without distant metastases. For this patient, the tumor burden reached a point where salvage chemotherapy and radiation therapy were no longer useful due to the involvement of the brachial neurovascular bundle. Therefore, forequarter amputation was used. As this case indicates, in cases where limb-sparing surgical resection is not feasible, forequarter amputations not only play a role in relieving upper limb pain, but can provide a chance for a cure. Although this is a major amputation and can have significant associated morbidity, a dedicated multidisciplinary team can help to reduce complications and achieve a successful rehabilitation.

## Consent

Written informed consent was obtained from the patient for publication of this case report and any accompanying images. A copy of the written consent is available for review by the Editor-in-Chief of this journal.

## Abbreviations

CT: Computed tomography
MRI: Magnetic resonance imaging
PET: Positron emission tomography.
